# Inflammatory bowel disease, such as Ulcerative colitis, is a risk factor for recurrent thromboembolic events: a case report

**DOI:** 10.1186/1757-1626-2-173

**Published:** 2009-10-29

**Authors:** Mortimer B O'Connor, Neil O'Donovan, Mark J Phelan, Michael J Regan

**Affiliations:** 1 Department of Medicine, South Infirmary - Victoria University Hospital, Old Blackrock Road, Cork, Ireland; 2 Department of Radiology, South Infirmary - Victoria University Hospital, Old Blackrock Road, Cork, Ireland; 3 Arthritis and Osteoporosis Centre, Department of Rheumatology, South Infirmary - Victoria University Hospital, Old Blackrock Road, Cork, Ireland; 4 The School of Medicine, University College Cork, Cork, Ireland

## Abstract

Ulcerative colitis (UC), a member of the family of inflammatory bowel disease (IBD), occurs worldwide. It has an incidence which in recent years has been rising in areas such as Southern Europe and Asia, while remaining relatively constant in Northern Europe and North America.

Complications associated with UC include toxic dilatation, perforation, carcinoma and massive haemorrhage. Extra-intestinal complications also occur and these include Thromboemboli (TE).

Thromboembolic disease has a greater incidence and recurrence rate among patients with IBD than the general population.

## Introduction

Ulcerative colitis (UC), a member of the family of inflammatory bowel disease (IBD), occurs worldwide. In the United Kingdom, the incidence of UC is approximately 10/100,000 with a point prevalence of 200/250,000 [1]. Presentation can occur at any age; however the typical age is in the third to fourth decade. Men and women tend to be equally affected. The incidence rates for UC have remained relatively constant in many regions such as Northern Europe and North America; however areas where there was previously a low incidence, such as Southern Europe and Asia, are showing an increase [1].

Patients with UC can suffer typically from diarrhoea, rectal bleeding and colicky abdominal pain. Symptoms most frequently present insidiously but may also present acutely, mimicking an infective aetiology. Its diagnosis is principally based on clinical, endoscopic and histological examinations [2].

Increasing data provide evidence of a complex interplay between components of the innate immune system and environmental factors, notably the microflora of the intestinal mucosa, in the healthy gut. Unusually smoking appears to be a protective in both development and exacerbations [3].

Complications associated with UC include toxic dilatation, perforation, carcinoma and massive haemorrhage. These are more common than in Crohn's disease. Extra-intestinal complications also occur and these include Thromboemboli (TE). The association between TE and IBD was first described by Bargen and Barker in 1936 [4].

Here we present the case of a patient with UC who has had recurrent TE.

## Case Report

A 61 year old gentleman presented to our Accident and Emergency department, via General Practitioner referral, complaining of a left below knee swelling, pain and superficial thrombophlebitis. The superficial thrombophlebitis had been present for seven days while the swelling and pain was of one day duration. The thrombophlebitis was unsuccessfully treated with amoxicillin 250 mg PO TDS daily along with diclofenac sodium 75 mg PO BD for pain relief.

In the week prior to this presentation he suffered an exacerbation of UC.

His past history included: Ulcerative colitis (diagnosed Aug 2005), pulmonary embolism (Oct 2005), Ischaemic heart disease, myocardial infarction with stenting to the long anterior descending artery (2003), hypercholesterolemia, hypertension and asthma. Medications on admission included: co-amoxiclav 625 mg PO BD × 4/7, olsalazine sodium 250 mg PO TDS, aspirin 75 mg PO OD, bumetanide 2 mg PO OD, rosuvastain 10 mg PO OD, bisoprolol fumarate 5 mg PO OD, amlodipine 10 mg PO OD, ramipril 10 mg PO OD, pantoprazole 40 mg PO OD, ergocalciferol one tablet POBD, salbutamol 100 mcg 2 puffs INH QDS and beclometasone dipropionate 250 mg two puffs INH BD. He had no known drug allergies.

The pulmonary emboli experienced in 2005 were diagnosed one week following an exacerbation on UC. At that presentation the C Reactive protein (CRP) was 102 mg/l, Erythrocyte sedimentation rate (ESR) 119 mm/h and D-dimer 2044 ng/ml. All other blood tests, which included a Factor V Leiden screen, were normal. Computed Tomography Pulmonary Angiogram demonstrated multiple emboli in both lungs.

On examination at his most recent presentation, he had a hot, swollen and tender left below knee swelling which was painful to touch on the medial aspect and in the popliteal fossa. The remainder of his examination was normal.

Investigations showed a raised D-dimer level of 4880 ng/ml, ESR 47 mm/h, CRP 7.1 mg/l. All other blood results being normal. A venogram confirmed the presence of a Deep Vein Thrombosis (DVT).

After a period of four days he was discharged on warfarin sodium, the dosage as per International normalized ratio (INR), along with all his medications at admission. The target INR was between 2 and 3. Follow up in out-patients clinic for INR level monitoring was arranged.

## Discussion

Patients with IBD are at an increased risk of thromboembolic complications by approximately three-fold [5], and both venous and arterial systems can be involved [6]. Studies using different approaches have revealed conflicting data on the prevalence of TE in IBD, varying between 1.2% and 6.7% in clinical studies and rising to 39% in postmortem studies [7]. In UC the incidence rate of DVT has been recorded as 30.0/10,000 person-years and for Pulmonary Emboli the value is 19.8/10,000. These values give an incident rate ratio to the general population of 2.8 (95% CI, 2.1 - 3.7) for DVT and 3.6 (95% CI, 2.5 - 5.2) for PE in patients with UC [8].

Despite the risk of thromboembolic disease (TED) in UC being recognised in adults, for over seventy years [4], and also in children [9], the exact pathogenetic mechanisms are unclear. Fluctuations in the levels of fibrinogen [10], factor II, V, and VIII [10], and fibrinopeptide A [10] have all been linked with active IBD. Active IBD has been recorded concurrently in up to 89% of cases [11]. Thrombosis, vasculitis, and tissue infarction have been proposed as contributing factors in UC and its exacerbations [10]. Furthermore, patients with haemophilia and Von Willebrand's disease have been shown to have a lower risk of IBD [11].

With an over all TE prevalence reported in the region of 3% [12], identification and prevention of such events is highly desirable as they are an important cause of morbidity and mortality in IBD patients. Among those with IBD who have a TE the mortality rate is up to 22% at a 1.8 year follow up [13]. The risk of death for UC patients is highest in the first months after a TE and then returns toward the rate for the general population [14]. This is compounded by the literature stating that the risk of recurrence of a TE is in the region of 13% [11]. Also of note 11% of TE in patients with IBD occur in unusual sites such as inominate veins, intracardiac sites and cerebral sites [11].

The published literature also states that 60% of TEs in UC occur in those under 50 years of age, which is younger than the general population [11] and females are more likely to suffer from TE events than males [14].

**Figure 1:**
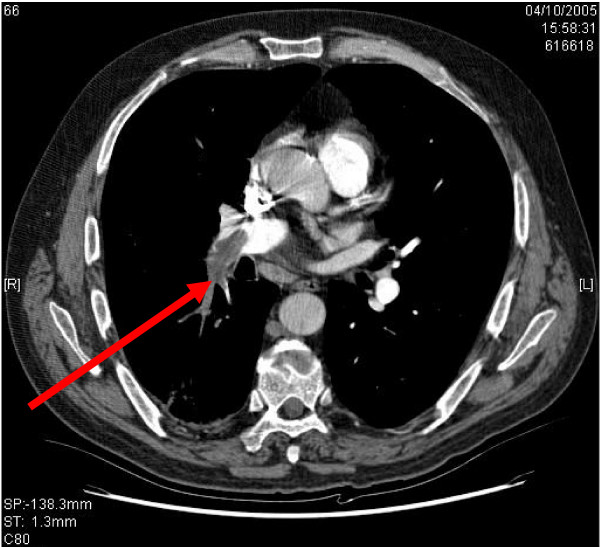
**A Computed Tomography Pulmonary Angiogram showing the filling defect associated with a pulmonary embolism (red arrow)**.

**Figure 2:**
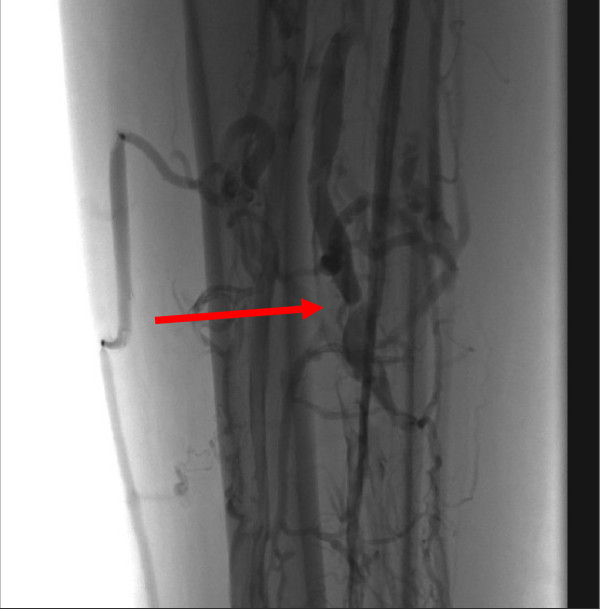
**A venogram of the lower limb showing a filling defect associated with a deep vein thrombosis (red arrow)**.

**Figure 3:**
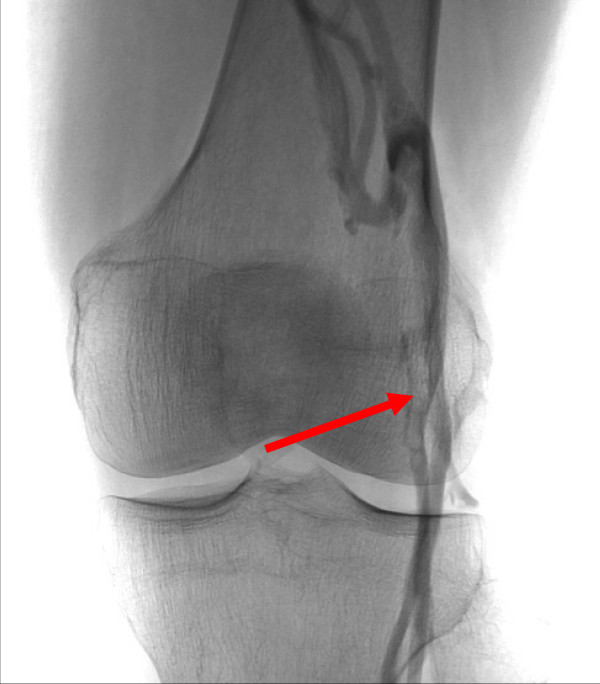
**A venogram of the lower limb showing a filling defect associated with a deep vein thrombosis (red arrow)**.

For those IBD patients with a TED history the long-term treatments are: 1. Anticoagulation alone, 2. Anticoagulation with an IVC filter and 3. IVC filter alone. However before a TE can be treated it must first be identified and imaging plays a major role in this process. In patients with UC, imaging for PE involves two modalities: Computed Tomography Pulmonary Angiogram and ventilation perfusion scanning. Regards imaging for DVT in patients with UC, venography and Doppler ultrasound are the predominant modalities. Figures 1, 2 &3 show radiological imaging on this patient.

## Conclusion

TED has a greater incidence among patients with IBD than the general population.

There is a 13% recurrence risk of a TE in the IBD population, while 11% experience their TE in an unusual site such as an intracardiac site.

A period of exacerbation of IBD is the time of greatest risk for a TE in a patient with an IBD such as UC.

## Consent

Written informed consent was obtained from the patient for publication of this case report and accompanying images. A copy of the written consent is available for review by the Editor-in-Chief of this journal.

## Competing interests

The authors declare that they have no competing interests.

## Authors' contributions

MOC interviewed and managed the patient care, reviewed the radiology and prepared the manuscript. NOD reviewed the radiology. MJP prepared the manuscript. MJR interviewed and managed the patient care, and prepared the manuscript.
